# Lipoprotein(a) and Atherosclerotic Cardiovascular Disease: Where Do We Stand?

**DOI:** 10.3390/ijms25063537

**Published:** 2024-03-21

**Authors:** Georgios Tsioulos, Dimitris Kounatidis, Natalia G. Vallianou, Aikaterini Poulaki, Evangelia Kotsi, Gerasimos Socrates Christodoulatos, Dimitrios Tsilingiris, Irene Karampela, Alexandros Skourtis, Maria Dalamaga

**Affiliations:** 1 Fourth Department of Internal Medicine, Medical School, University General Hospital Attikon, National and Kapodistrian University of Athens, 12462 Athens, Greece; geotsioulos@med.uoa.gr; 2 Second Department of Internal Medicine, School of Medicine, Hippokration General Hospital, National and Kapodistrian University of Athens, 11527 Athens, Greece; dimitriskounatidis82@outlook.com (D.K.); lila.kotsi@yahoo.com (E.K.); 3 First Department of Internal Medicine, Sismanogleio General Hospital, 15126 Athens, Greece; natalia.vallianou@hotmail.com; 4 Hematology Unit, Second Department of Internal Medicine, School of Medicine, National and Kapodistrian University of Athens, 11527 Athens, Greece; aikaterini.poulaki@gmail.com; 5 Department of Microbiology, Sismanogleio General Hospital, 15126 Athens, Greece; gerchristod82@hotmail.com; 6 First Department of Internal Medicine, University Hospital of Alexandroupolis, Democritus University of Thrace, 68100 Alexandroupolis, Greece; tsilingirisd@gmail.com; 7 Second Department of Critical Care, Attikon General University Hospital, Medical School, National and Kapodistrian University of Athens, 12462 Athens, Greece; eikaras1@gmail.com; 8 Department of Internal Medicine, Evangelismos General Hospital, 10676 Athens, Greece; alex.skourtis@gmail.com; 9 Department of Biological Chemistry, Medical School, National and Kapodistrian University of Athens, 11527 Athens, Greece

**Keywords:** antisense oligonucleotides, atherosclerosis, cardiovascular disease, chronic inflammation, lepodisiran, lipoprotein(a), muvalaplin, small interfering RNAs

## Abstract

Lipoprotein(a) [Lp(a)] consists of a low-density lipoprotein-like molecule and an apolipoprotein(a) [apo(a)] particle. Lp(a) has been suggested to be an independent risk factor of atherosclerotic cardiovascular disease (ASCVD). Lp(a) plasma levels are considered to be 70–90% genetically determined through the codominant expression of the *LPA* gene. Therefore, Lp(a) levels are almost stable during an individual’s lifetime. This lifelong stability, together with the difficulties in measuring Lp(a) levels in a standardized manner, may account for the scarcity of available drugs targeting Lp(a). In this review, we synopsize the latest data regarding the structure, metabolism, and factors affecting circulating levels of Lp(a), as well as the laboratory determination measurement of Lp(a), its role in the pathogenesis of ASCVD and thrombosis, and the potential use of various therapeutic agents targeting Lp(a). In particular, we discuss novel agents, such as antisense oligonucleotides (ASOs) and small interfering RNAs (siRNAs) that are currently being developed and target Lp(a). The promising role of muvalaplin, an oral inhibitor of Lp(a) formation, is then further analyzed.

## 1. Introduction

Low-density lipoprotein (LDL) particles serve as the primary carriers of cholesterol in circulation. Each LDL particle comprises a lipid core primarily composed of cholesteryl ester (CE) molecules, along with small amounts of triglycerides (TG) and unesterified cholesterol (UC). The surface of the LDL particle is enveloped by a monolayer consisting of approximately 700 phospholipid molecules and a single copy of apolipoprotein B100 (ApoB100) [1]. ApoB100 is also the predominant apolipoprotein found in very low-density lipoproteins (VLDLs), intermediate-density lipoproteins (IDLs), and lipoprotein(a) particles. Lp(a) is a specific subtype of low-density lipoprotein cholesterol (LDL-C), distinguished by the covalent attachment of ApoB100 to a unique glycoprotein known as apolipoprotein(a). The lipid composition of the LDL moiety within Lp(a) closely resembles that of LDL-C itself. However, apo(a) features several triple-loop structures called “kringles,” which are also present in coagulation factors such as plasminogen and prothrombin. These kringle domains play a crucial role in apo(a)’s properties, including its interactions with receptors on vascular and inflammatory cells, as well as with fibrin [2].

Since its first description by Kare Berg in 1963 [3], Lp(a) has undergone extensive investigation, mainly due to its role in atherosclerosis and cardiovascular disease. Lp(a) synthesis takes part almost exclusively in the liver through the coupling of LDL and apo(a) molecules, while the precise paths of this process remain obscure [4]. Lp(a) is a well-established independent risk factor for ASCVD and calcific aortic valvular disease (CAVD) [5]. Current literature supports that Lp(a)’s involvement in the pathogenesis of ASCVD is attributed to its proatherogenic, proinflammatory, and prothrombotic properties, which appear to be more potent than those of LDL-C [3]. It is estimated that over 1.4 billion people worldwide have elevated levels of Lp(a), defined as above 50 mg/dL or 125 nmol/L, accounting for approximately 20–25% of the world’s population. A recent multicenter, cross-sectional, epidemiological study, which included patients with established ASCVD from 48 countries worldwide, demonstrated that more than 25% of these patients had Lp(a) levels exceeding the aforementioned threshold for increased cardiovascular risk [6,7]. Moreover, Lp(a) is under strong genetic control, since approximately up to 90% of Lp(a) levels are inherited and genetically determined by a gene encoding its apo(a) component, namely, the *LPA* gene [4]. Lp(a)’s strong genetic component implies relatively constant serum levels throughout an individual’s life. Hence, current guidelines support once-in-a-lifetime measurement in most individuals with an increased risk of ASCVD [5].

Several lipid-modifying therapies have been explored for their potential to reduce lipoprotein(a) levels, with varying degrees of success. Among them, proprotein convertase subtilisin/kexin 9 (PCSK9) inhibitors have emerged as a promising approach for reducing both Lp(a) levels and the risk of major adverse cardiovascular events (MACEs) [8]. Recent advancements in RNA interference (RNAi) technology have opened up new perspectives for developing treatments targeting apo(a) messenger ribonucleic acid (mRNA) degradation prior to translation. Potential therapeutic approaches involve nucleic acid therapeutics, which are designed to specifically target gene expression and can be delivered via ASOs or siRNA molecules [9]. In addition to nucleic acid-based therapies, muvalaplin, a small molecule inhibitor of Lp(a) particle formation, has shown promising results in reducing serum Lp(a) levels. Unlike ASOs and siRNAs, muvalaplin can be administered orally and acts by disrupting the noncovalent interaction between ApoB100 and apo(a), specifically binding to the apo(a) kringle KIV7 and KIV8 domains [10]. Although these novel therapeutic interventions have demonstrated significant efficacy in reducing Lp(a) levels, their impact on cardiovascular risk remains uncertain.

The aim of this review is to summarize recent evidence regarding the structure, metabolism, and factors affecting the circulating levels of Lp(a), as well as the laboratory determination of Lp(a), its role in the pathogenesis of ASCVD and thrombosis, and the potential use of various drugs targeting Lp(a). Special emphasis will be given to novel therapeutic agents, such as antisense oligonucleotides and small interfering RNAs, which are currently being developed and target Lp(a). The promising role of muvalaplin, an oral inhibitor of Lp(a) formation will be further analyzed.

## 2. Literature Search

For the preparation of this narrative review, we conducted a search in the PubMed NIH database using the search terms “Lp(a)” and “atherosclerosis”. Our search was limited to items published within the past 10 years, yielding a total of 738 outputs from 2014 to February 2024. We focused on research and review articles, randomized clinical trials, and meta-analyses. Additionally, we reviewed the references of these articles to identify other relevant publications. Given the extensive number of manuscripts retrieved, it is acknowledged that not all of them can be covered comprehensively within the scope of this review.

## 3. Lipoprotein(a): Structure, Synthesis, and Metabolism

**Figure 1:**
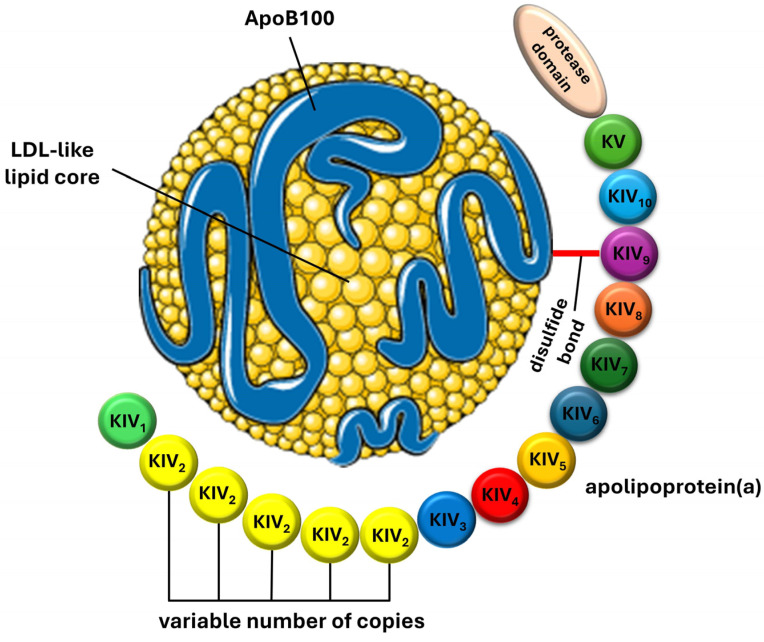
Structure of lipoprotein(a). Lipoprotein(a) is a variant of LDL, distinguished by the binding of ApoB100 to apolipoprotein(a) via a disulfide thioester bond. Abbreviations: ApoB100, Apolipoprotein B100; KIV, kringle IV. LDL, low-density lipoprotein. Parts of the figure are from the free medical site http://smart.servier.com/ (accessed on 10 March 2024) by Servier licensed under a Creative Commons BY 4.0 License https://creativecommons.org/licenses/by/4.0/ (accessed on 10 March 2024).

Lipoprotein(a) is a variant of LDL-C, distinguished by the covalent binding of ApoB100 to a unique glycoprotein called apolipoprotein(a) via a disulfide thioester bond. This structural difference results in variations in molecular weight, density, and electrophoretic mobility compared to LDL-C due to the presence of apo(a) [11]. Apo(a) is highly polymorphic and comprises variable numbers of cysteine-rich domains known as kringles. Kringles are triple-loop structures stabilized by internal disulfide bonds and are also found in coagulation factors such as plasminogen, prothrombin, urokinase, and tissue-type plasminogen activators [12]. While plasminogen contains five kringle domains (KI, KII, KIII, KIV, and KV) and one protease domain, apo(a) consists of a single kringle V domain (KV), ten different types of kringle IV domains (KIV1 to KIV10), and a catalytically inactive protease domain at the carboxyl terminus [13]. The size heterogeneity of apo(a) is determined by the variable number of KIV2 domain copies, which can range from 1 to over 40, while the remaining KIV domains are typically present as single copies [14,15]. Figure 1 provides a schematic presentation of the structure of the Lp(a) particle.

Lp(a) synthesis primarily occurs in the liver, although small amounts of apo(a)-mRNA have been detected in various tissues including the brain, lungs, testes, pituitary, and adrenal glands [4]. The assembly of Lp(a) involves two consecutive steps [16]. In the first step, lysine residues at the N-terminus of ApoB100 are noncovalently bound to lysine-binding sites located at the KIV7 and KIV8 domains of apo(a). Subsequently, in the second step, a covalent disulfide bridge is established between cysteine residues in the KIV9 of apo(a) and ApoB100 [17]. The exact location of this assembly process remains uncertain. Recent evidence suggests that the first step occurs intracellularly, while the second step takes place extracellularly [18,19].

The clearance of Lp(a) molecules primarily occurs in the liver, with a small fraction also removed by the kidneys [20]. Additionally, the spleen and muscles may play a minor role in this process [21]. Lp(a) clearance involves various cell surface receptors, including the LDL receptor (LDL-R), scavenger receptors, various plasminogen receptors, Toll-like receptors (TLRs), and carbohydrate receptors or lectins. However, the exact role and degree of involvement of each receptor remain uncertain [17,22]. ApoB100, apo(a), and oxidized phospholipids (OxPLs) on the surface of Lp(a) act as ligands for these receptors [23]. Intracellularly, LDL and apo(a) particles undergo lysosomal degradation, while approximately 30% of apo(a) molecules are recycled to contribute to the formation of new Lp(a) molecules [24]. The relatively similar rate of fractional catabolism among different sizes of apo(a) isoforms ensures that the catabolism rate of Lp(a) remains relatively constant, thus not significantly affecting its plasma concentration [25].

## 4. Factors Affecting the Lp(a) Levels: Genetics and beyond

### 4.1. Genetics

Serum Lp(a) concentration displays wide variation among individuals, with up to 90% of Lp(a) levels being inherited and genetically determined by the *LPA* gene [4]. This gene, structurally homologous to plasminogen (PLG), encodes the apo(a) component of Lp(a) [12]. The genetically predetermined number of KIV2 copies on the *LPA* locus, which determines the size of different apo(a) isoforms, explains approximately 30 to 70% of the variability in the Lp(a) concentration [5]. There exists an inverse relationship between the number of KIV2 copies and plasma Lp(a) levels, where fewer KIV2 copies result in smaller apo(a) isoforms and higher rates of Lp(a) secretion. On the contrary, a higher number of KIV2 copies accounts for larger apo(a) isoforms and lower Lp(a) levels [15,26,27]. This phenomenon is attributed to the susceptibility of larger apo(a) molecules to proteasomal degradation within hepatocytes [28]. Every individual carries two copies of the *LPA* gene located on chromosome 6, with one copy on each allele. Serum Lp(a) levels reflect the combined effects of apo(a) isoforms produced by each allele [29]. Consequently, the allele encoding the smaller apo(a) isoform predominantly determines the main isoform in an individual [30].

In addition to the variability in apo(a) isoform size, genetic variants play a crucial role in determining Lp(a) concentration [31]. Several independent single nucleotide polymorphisms (SNPs) located around the *LPA* gene are among the most important determinants of Lp(a) levels [32]. Some of these genetic variants lead to decreased Lp(a) levels. For example, common splice variants such as 4925G>A and 4733G>A in the KIV region, carried by approximately 38% and 22% of the population respectively, contribute to lower Lp(a) levels [33,34]. Additionally, missense variants like rs41267813 also lead to reduced Lp(a) concentrations [35]. On the contrary, genetic variants, such as rs1800769 and rs1853021, are associated with higher Lp(a) levels [36]. Interestingly, rs10455872 and rs3798220 variants have been further suggested to correlate with the increased risk of coronary heart disease (CHD) [37]. Moreover, certain genetic variants, such as rs41272114, rs41259144, and rs139145675, lead to nonfunctional (null) alleles, which are associated with a protective effect against the risk of ASCVD [15,38].

While the *LPA* gene region plays a significant role in determining Lp(a) levels, other genes outside of this region may also contribute to Lp(a) concentration regulation [31]. Although initial studies did not identify candidate genes outside the *LPA* locus affecting Lp(a) levels, more recent research suggests otherwise [39]. A genome-wide association study conducted on approximately 300,000 individuals from the UK Biobank identified additional loci that influence Lp(a) concentration. Specifically, genes such as *APOE*, *CETP*, and *APOH* were found to be determinants of Lp(a) levels, indicating a broader genetic influence on Lp(a) regulation [40]. Furthermore, genetic disorders affecting lipoprotein metabolism might have varying effects on Lp(a) levels. Conditions such as abetalipoproteinemia, lecithin-cholesterol acyltransferase (LCAT) deficiency, and lipoprotein lipase deficiency are associated with decreased Lp(a) levels. In contrast, familial hypercholesterolemia (FH) and familial defective ApoB100 (FDB) are characterized by increased Lp(a) levels alongside elevated levels of other lipoproteins [41,42].

### 4.2. Beyond Genetics

#### 4.2.1. Age, Gender, and Ethnicity

Serum Lp(a) concentration exhibits relative stability over an individual’s lifespan due to its strong genetic influence. By the age of 2, the genes responsible for Lp(a) synthesis typically reach full expression, and, by around the age of 5, the final adult concentration is generally attained, although levels may continue to rise until adulthood [5,43]. Furthermore, serum Lp(a) levels provide significant variation among different ethnic groups [31]. In a large observational study involving 4732 adults from the Atherosclerosis Risk in Communities (ARIC) study, the absolute change in Lp(a) concentration over a 15-year period was generally modest for most individuals. Participants were categorized based on their baseline Lp(a) concentrations into three groups: normal (<30 mg/dL), borderline-high (30–49 mg/dL), or high (≥50 mg/dL). Traditionally, two cutoff values for Lp(a) levels, namely, 30 mg/dL and 50 mg/dL, have been utilized to identify individuals at higher risk of ASCVD, with 50 mg/dL representing approximately the 80th percentile in the populations studied. Notably, individuals with high baseline concentrations experienced greater changes over time. This study suggested that adults with borderline high Lp(a) concentrations, particularly those who are Afro-Americans, female, or have comorbidities such as diabetes, arterial hypertension, or albuminuria, may benefit from repeated measurements of Lp(a) over time [44].

Data from the UK Biobank indicate a sequential increase in median Lp(a) levels among individuals of Chinese, White, South Asian, and Afro-American descent [45,46]. Similarly, the ARIC study demonstrated wider variation in Lp(a) levels among Afro-American individuals compared to White individuals [47]. Notably, except for Afro-Americans and individuals from India, most ethnicities show a skewed distribution of serum Lp(a) levels towards lower values [48]. In the Dallas Heart Study, the inverse correlation between KIV2 copies and Lp(a) levels was observed across Afro-Americans, White, and Hispanic individuals, with Afro-Americans generally exhibiting higher Lp(a) concentrations for a given number of KIV2 repeats [49]. While earlier studies provided conflicting evidence, recent research suggests a gender difference in Lp(a) levels, with women typically having 5 to 10% higher levels than men [46,50,51,52,53]. Furthermore, while Lp(a) levels tend to remain stable throughout men’s lifetime, women may experience an increase in levels after menopause [54]. These gender-related differences appear consistent across various racial groups [55].

#### 4.2.2. Liver and Kidney Disorders

Liver disease can lead to a reduction in plasma Lp(a) levels, as the liver is the primary site of Lp(a) synthesis [55]. Interestingly, in liver transplant recipients, there is a shift in apo(a) isoforms to those of the donor, resulting in changes in Lp(a) concentration [56]. Conversely, chronic kidney disease and nephrotic syndrome have been associated with increased Lp(a) levels. This increase is attributed to either reduced catabolism or increased hepatic production in response to protein loss in urine or during dialysis [57]. Notably, kidney transplantation has been shown to restore Lp(a) levels to their original values within a few weeks [58].

#### 4.2.3. Hormones

Various hormonal changes may also influence Lp(a) levels [59]. Conditions such as hypothyroidism, growth hormone deficiency in adults, and the depletion of endogenous sex hormones (e.g., menopause, ovariectomy, castration, orchidectomy) have been associated with increased Lp(a) concentration [60,61,62,63]. Conversely, hormonal replacement therapy with thyroxine or in cases of hyperthyroidism, as well as hormonal replacement treatment in postmenopausal women, have been shown to reduce Lp(a) levels in a case-dependent manner [64,65,66]. However, the impact of this reduction on cardiovascular disease (CVD) risk remains a topic of debate [67].

#### 4.2.4. The Role of Inflammation

**Figure 2:**
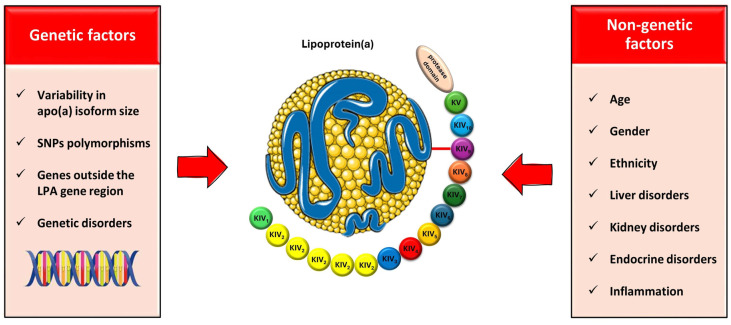
Genetic and non-genetic factors affecting lipoprotein(a) levels. Abbreviations: Apo(a), Apolipoprotein(a); KIV, kringle IV; SNPs, single nucleotide polymorphisms. Parts of the figure are from the free medical site http://smart.servier.com/ by Servier licensed under a Creative Commons BY 4.0 License https://creativecommons.org/licenses/by/4.0/).

Serum Lp(a) levels are influenced by inflammatory states in various ways. The presence of interleukin-6 (IL-6) response elements in the *LPA* gene suggests that Lp(a) may act as an acute phase reactant in inflammatory conditions, including autoimmune diseases and myocardial infarction (MI) [68,69,70,71,72]. The IL-6 receptor blockade following tocilizumab injection has been shown to effectively reduce Lp(a) levels [73]. Additionally, Lp(a) levels appear to have increased during the Coronavirus disease 2019 (COVID-19) infection, potentially contributing to the increased thromboembolic risk associated with the disease [74]. Interestingly, in life-threatening conditions such as sepsis or severe burns, there is a significant reduction in serum Lp(a) levels, suggesting a possible role of Lp(a) as a negative acute phase reactant [75]. It is important to recognize that serum Lp(a) levels are influenced by coexisting inflammatory or medical conditions and should be interpreted in the context of these factors. The main factors influencing Lp(a) levels are summarized in Figure 2.

## 5. Lp(a) Measurement and Reporting: Current Knowledge and Concerns

The measurement of Lp(a) concentration presents challenges due to its structural complexity, lipid composition variations, and the diverse sizes of apo(a) isoforms [76]. Many commercial immunoassays utilize polyclonal antibodies that may cross-react with different numbers of KIV2 copies, leading to the potential overestimation or underestimation of Lp(a) levels based on the apo(a) isoform size [77]. A study comparing six commercially available immunoassays revealed significant discrepancies among them, highlighting the need for improved standardization [78]. A newly developed latex-enhanced immunoturbidimetric assay shows promise in mitigating the impact of apo(a) isoform size differences compared to traditional enzyme-linked immunosorbent assays (ELISAs) [79]. Additionally, a liquid chromatography–tandem mass spectrometry (LC-MS/MS) assay has emerged as a potentially superior method unaffected by apo(a) isoform size polymorphism, making it a candidate for standardizing Lp(a) measurements [80]. This advancement holds promise for more accurate and consistent assessments of Lp(a) levels in clinical practice.

Despite challenges in measuring Lp(a) levels, reporting them accurately is equally important. Currently, there are two main methods for reporting Lp(a) levels. The first method involves reporting total Lp(a) mass concentrations, which include the mass of apo(a), ApoB100, lipid, and carbohydrate components. These values are typically expressed in mg/dL. However, there is a lack of traceability from the various calibrators to the reference materials, which can affect the consistency and comparability of results [81]. In contrast, the second method reports Lp(a) particle numbers in molar concentration units, expressed as nmol/L of apo(a). This method utilizes assay calibrators that are traceable to the World Health Organization/International Federation of Clinical Chemistry and Laboratory Medicine (WHO/IFCCLM) secondary reference material. This approach offers comparability to the “gold standard” monoclonal, antibody-based ELISA method [79,82].

Current recommendations advocate for using assays least affected by varying apo(a) isoform sizes and calibrated with WHO/IFCCLM reference material. Additionally, assays reporting Lp(a) particle numbers in molar concentration units (nmol/L) are preferred over those reporting mass units (mg/dL). However, if assays reporting particle numbers are unavailable, using the units in which the assay is calibrated is recommended for reporting. This ensures greater accuracy and standardization of Lp(a) measurements across different laboratories and methods [83,84].

## 6. The Role of Lp(a) in Atherosclerosis

The effects of Lp(a) on cardiovascular disease are thought to be related to its pro-atherogenic, proinflammatory, and prothrombotic properties, which have primarily been demonstrated through in vitro studies. Lp(a) plays a key role in the pathophysiology of atherosclerosis [2]. Similar to other atherogenic lipoproteins, the arterial influx of Lp(a) depends on factors such as plasma concentration, arterial wall permeability, and arterial blood pressure [85]. Lp(a) particles anchor to the exposed surface of denuded endothelium or aortic valve leaflets through its lipoprotein structure and lysine binding sites of apo(a), facilitating attachment to the extracellular matrix [86,87,88]. Once inside the vessel wall, Lp(a) molecules primarily accumulate extracellularly in the intima and subintima, with some integration into cells, particularly macrophages that transform into foam cells [89,90]. This conversion is mediated by a combination of intracellular and extracellular interactions that promote lipid-driven atherogenesis [91]. Notably, despite its lower serum concentration compared to LDL-C, Lp(a) preferentially accumulates at sites of injured endothelium [90].

Lp(a) provokes atherogenesis by promoting the recruitment of inflammatory cells into the vessel wall [3]. It upregulates adhesion molecules such as vascular cell adhesion molecule-1 (VCAM-1), E-selectin, intercellular adhesion molecule-1 (ICAM-1), and β2-integrin macrophage-1 (Mac-1), facilitating the attachment and infiltration of monocytes [92]. Lp(a) also induces the production of chemokines, either directly through apo(a) lysine binding sites or indirectly by stimulating endothelial cells to secrete monocyte chemoattractant protein (MCP) [93]. Furthermore, Lp(a) facilitates the release of IL-8 by macrophages through oxidized phospholipids bound to apo(a), thereby enhancing neutrophil infiltration [94,95]. Additionally, Lp(a) increases the expression of proinflammatory cytokines, such as IL-1β and tumor necrosis factor-α (TNF-α), through macrophages, further exacerbating inflammation in the arterial wall [96,97].

The role of OxPLs in the early stages of atherosclerosis is well-documented, featuring the oxidative modulation of lipoproteins, including Lp(a), and generating mediators that influence inflammation [98]. OxPLs present on oxidized LDLs (OxLDLs) can initiate sterile inflammation, thereby triggering a cascade of atherosclerosis [99]. Notably, approximately 90% of oxidized phospholipids in human lipoproteins are carried by Lp(a), emphasizing its potential role in binding, and transporting serum OxPLs. [100].

Lp(a) particles also carry significant amounts of lipoprotein-associated phospholipase A2 (Lp-PLA2), which hydrolyzes OxPLs. This suggests a possible role of Lp(a) in scavenging atherogenic phospholipids from the circulation and mediating their clearance [101]. However, higher concentrations of Lp(a) can lead to the excessive accumulation of OxPLs in the arterial wall, contributing to atherosclerosis [102]. Among the most important atherogenic actions of Lp(a) are the endothelial dysfunction, proliferation, and migration of vascular smooth muscle cells (VSMCs) into atheromatous plaques, generation of reactive oxygen species, chemotaxis, formation of foam cells, inflammation, and plaque instability. OxPLs, carried by Lp(a), contribute to these actions similarly to LDL molecules [103]. Furthermore, Lp(a) plays a significant role in inducing the destabilization of atherosclerotic plaques. Enzymes detected in atherosclerotic sites, such as matrix metalloproteinases (MMPs) and elastases, split Lp(a) molecules into fragments, with some fragments interacting with proteins involved in atherogenic effects [104,105]. OxPLs bound to apo(a) enhance the apoptosis of endoplasmic reticulum-stressed macrophages, further contributing to plaque necrosis [106]. The aforementioned mechanisms collectively underscore the multifaceted and complex role of Lp(a) in the pathogenesis of atherosclerosis.

## 7. The Role of Lp(a) in Cardiovascular Disease: Focusing on Atherothrombosis

### 7.1. The Role of Lp(a) in Atherosclerotic Cardiovascular Disease (ASCVD)

Genetic, experimental, and observational data consistently identify Lp(a) as an independent risk factor for ASCVD, aortic valve stenosis (AVS), and cardiovascular mortality in both men and women, spanning various ethnic groups [5]. This correlation is particularly pronounced for myocardial infarction, stroke, atherosclerotic stenosis, and AVS [107,108,109]. In a study conducted on individuals with MI/CHD and controls as part of the Reykjavik Study, researchers observed a significant association between elevated levels of Lp(a) and the risk of CHD. Specifically, individuals in the top tertile (highest third) of Lp(a) levels had a substantially increased risk of CHD compared to those in the lower tertiles [110]. Elevated levels of Lp(a) have also been associated with recurrent cardiovascular events, particularly when LDL-C levels are high. However, the nature of this correlation may change in subjects with extremely low LDL-C concentrations [111]. Furthermore, serum Lp(a) seems to remain elevated six months after an acute MI, and high levels are associated with a more severe clinical expression of coronary artery disease (CAD) [71,112].

Regarding cerebrovascular disease, a large-scale Danish study showed that elevated Lp(a) concentrations are associated with a higher incidence of ischemic stroke [108]. Interestingly, a large systematic review showed an approximately two-fold increase in the relative risk for ischemic stroke related to high Lp(a) concentrations [113]. Moreover, meta-analysis data suggest that high Lp(a) levels are associated with increased odds of cognitive impairment and disability related to stroke [114]. However, the impact of Lp(a) levels on peripheral artery disease (PAD) is less clear, with conflicting findings in the literature. While some studies report a direct association between Lp(a) concentrations and PAD, others have found conflicting results [115]. Recent research suggests that high Lp(a) levels may be a significant predisposing factor for PAD, particularly in female subjects [116].

Lp(a) has been identified as an independent predictor of carotid artery stenosis and occlusion [117]. Notably, carotid intima-media thickness (CIMT) serves as an ultrasound index for assessing CVD risk in both primary and secondary prevention, particularly among individuals with subclinical and asymptomatic CVD. Elevated CIMT values have been identified as predictors of future CVD events and cardiovascular mortality, as well as markers of response to hypolipidemic therapy. The American Society of Echocardiography (ASE) suggests that a CIMT value exceeding the 75th percentile should be considered pathological [118]. However, the 2021 ESC Guidelines on CVD prevention in clinical practice have discouraged the use of CIMT for assessing CVD burden due to concerns regarding methodological standardization [119]. Moreover, the relationship between Lp(a) levels and CIMT and carotid plaque formation remains contentious in the literature, albeit recent studies have attempted to address this issue. França et al. investigated the potential association between serum Lp(a) levels and subclinical atherosclerosis in 317 healthy individuals by measuring CIMT and assessing carotid plaque presence. They found that Lp(a) levels exceeding 30 mg/dL were significantly associated with carotid plaque formation, although CIMT was not influenced by serum Lp(a) concentrations [120]. Conversely, a large population cross-sectional study involving 411,634 healthy Chinese individuals reported that Lp(a) concentrations ≥50 mg/dL were associated with a higher prevalence of carotid atherosclerosis, as determined using CIMT measurements and an assessment of carotid plaques [121].

Mendelian randomization studies have further supported the association between genetic variants related to increased Lp(a) levels and the prevalence and incidence of cardiovascular events [41]. Conversely, variants associated with decreased Lp(a) concentrations have shown a protective effect against ASCVD [33,34,122]. While much of the research initially focused on White populations, data from studies such as the UK Biobank, ARIC, INTERHEART, and MESA have confirmed that the association between Lp(a) levels and ASCVD risk extends across different ethnicities [46,47,48,123].

Furthermore, epidemiological studies have demonstrated a continuous and linear correlation between serum Lp(a) levels and ASCVD risk, unaffected by a threshold effect [47,123,124]. Individuals with very high serum Lp(a) levels (>180 mg/dL or >430 nmol/L) are considered to have an equivalent lifetime ASCVD risk to those with untreated heterozygous familial hypercholesterolemia (HeFH) [125]. Notably, Lp(a) is recognized as an independent cardiovascular risk factor irrespective of LDL-C levels. This was highlighted in studies like JUPITER, FOURIER, and ODYSSEY-OUTCOMES, where residual ASCVD risk attributed to Lp(a) remained significant even in patients with low LDL-C levels, indicating that Lp(a)-related risk is distinct from that associated with LDL-C. [126,127,128]. Recent studies have highlighted a significant additional association between high-sensitivity C-reactive protein (hs-CRP) and Lp(a) levels as joint predictors of major adverse cardiovascular events (MACEs) [129].

### 7.2. The Role of Lp(a) in Calcific Aortic Vascular Disease (CAVD)

Calcific aortic valvular disease (CAVD), which encompasses both aortic valvular sclerosis and stenosis, represents the most prevalent heart valve disorder in developed countries. Despite its high prevalence, there is currently no available medical treatment for CAVD. However, recent research has shed light on the role of serum Lp(a) as an independent causal risk factor in the development of CAVD, highlighting its significance in the pathogenesis and progression of this common heart valve disorder [5,130]. Studies have identified Lp(a) as a key contributor to the pathogenesis of CAVD through a ‘’three hit’’ mechanism involving lipid deposition, inflammation, and the transport of autotaxin, an important enzyme used for generating the lipid-signaling molecule lysophosphatidic acid. These processes ultimately lead to the transition of valve interstitial cells into osteoblast-like cells and subsequent parenchymal calcification, a hallmark of CAVD progression [131]. Evidence supporting the association between elevated levels of Lp(a) and the risk of developing CAVD dates back to the mid-1990s [132]. More recent genetic research, including genome-wide association studies, has further strengthened this link by identifying specific genotypic variants in the *LPA* gene that are correlated with aortic valve calcification and stenosis [133]. These findings underscore the importance of Lp(a) in the pathophysiology of CAVD and suggest potential implications for the development of targeted therapeutic strategies aimed at mitigating the progression of this prevalent heart valve disorder.

Numerous cohort studies, case-control studies, and meta-analyses have subsequently validated and strengthened these initial findings, reinforcing the role of elevated Lp(a) levels in CAVD development [134,135,136,137]. Moreover, elevated Lp(a) levels have been linked not only to an increased risk of developing CAVD but also to faster disease progression, an earlier need for aortic valve replacement, and higher mortality rates [138,139]. Research studies suggest that elevated Lp(a) levels induce both the microcalcification and macrocalcification of the aortic valve, particularly affecting healthy individuals between 45 and 54 years of age [140]. Conversely, decreased Lp(a) levels are associated with a lower risk of aortic stenosis [141].

### 7.3. The Role of Lp(a) in Thrombosis

In vitro studies have demonstrated that Lp(a) can interfere with various stages of hemostasis, resulting in the inhibition of fibrinolysis. However, as of now, this apparent prothrombotic effect of Lp(a) has not been conclusively demonstrated in vivo [142,143]. Lp(a) may exert its effect on fibrinolysis due to the structural homology between the KIV domain of apo(a) and the fibrin-binding domain of plasminogen (*PLG*). This structural similarity suggests that there may be competition between apo(a) and *PLG* for fibrin affinity sites [144]. Moreover, research has shown that Lp(a) can attenuate the tissue plasminogen activator (tPA)-induced conversion of plasminogen to plasmin in the presence of fibrin. This inhibition occurs through the upregulation of plasminogen activator inhibitor-1 (PAI-1), a key inhibitor of fibrinolysis [145].

Several studies have provided evidence that Lp(a) plays a role in platelet activation and aggregation, particularly in response to certain agonists. While Lp(a) appears to play a role in arterial thrombosis and atherosclerosis-related events, its association with non-atherosclerotic thrombotic disorders such as venous thromboembolism (VTE) is less pronounced, suggesting distinct pathophysiological mechanisms underlying these conditions. Thus, despite its role in atherosclerosis and arterial thrombosis, Lp(a) does not appear to be a risk factor for some non-atherosclerotic thrombotic disorders such as VTE, deep vein thrombosis (DVT), and pulmonary embolism (PE) [145]. Observational studies have shown that the risk of VTE is only slightly increased in individuals with very high Lp(a) levels (greater than the 95th percentile). Mendelian association studies, which utilize genetic variants associated with Lp(a) levels as instrumental variables, have further supported these observations by demonstrating a lack of causality between Lp(a) and VTE [146].

## 8. The Role of Lp(a) beyond Atherosclerosis

**Figure 3:**
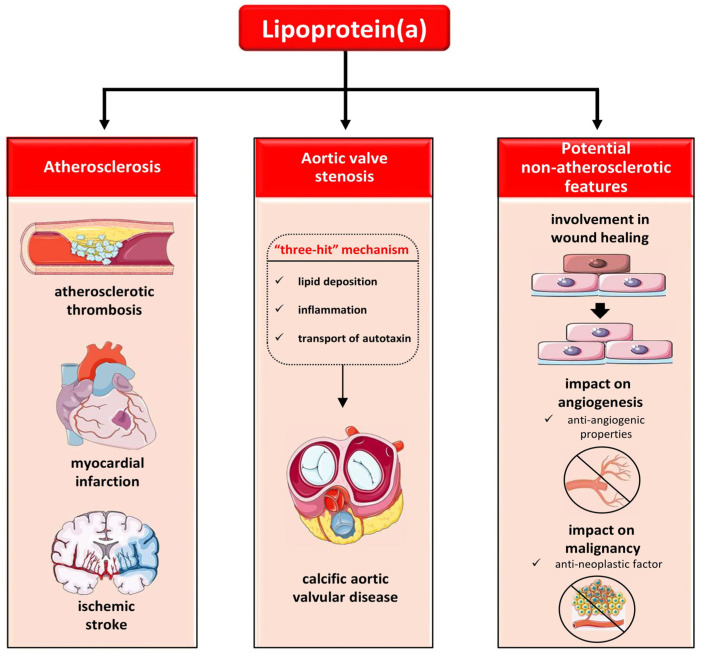
The role of lipoprotein(a) in human disease. Elements of the figure are from the free medical site http://smart.servier.com/ by Servier licensed under a Creative Commons BY 4.0 License https://creativecommons.org/licenses/by/4.0/.

The exact physiological role of Lp(a) in humans remains a topic of ongoing research and debate. Its potential involvement in wound healing is intriguing, given its ability to transport essential molecules and its accumulation at sites of endothelial barrier disruption [147,148]. Through its interactions with various components of the vessel wall and subendothelial matrix, Lp(a) can stimulate the activation of immune cells like monocytes/macrophages, trigger the hemostatic mechanism, and modulate angiogenesis. These effects are largely mediated by apo(a), the unique component of Lp(a) [149]. On the other hand, emerging evidence suggests that Lp(a) may possess anti-angiogenic properties. It seems to interfere with the activation of proteases essential for angiogenesis, such as MMPs [150,151,152]. This anti-angiogenic potential, combined with its structural similarity to plasminogen, raises the possibility of Lp(a) acting as an anti-neoplastic factor [31,153]. Given that inflammation and endothelial dysfunction are key drivers of the atherosclerotic process, the properties of Lp(a) related to wound healing and angiogenesis may provide insights into its role in atherosclerosis. However, further research is warranted to fully elucidate the mechanisms underlying these potential functions of Lp(a) and their implications for human health and disease. The role of lipoprotein(a) in human disease is illustrated in Figure 3.

## 9. The Impact of Lipid-modifying Interventions on Lp(a) Levels: From Traditional to Novel Agents

### 9.1. Fasting and Lifestyle Modifications

The impact of various lifestyle factors, including diet and exercise, on Lp(a) levels remains an area of ongoing research with some conflicting findings. Replacing dietary saturated fatty acids (SFAs) with other macronutrients, such as monounsaturated fatty acids (MUFAs) or carbohydrates, has been shown to increase Lp(a) concentrations while decreasing LDL-C levels [154,155,156]. Conversely, low carbohydrate/high saturated fat diets have been associated with an up to 15% decrease in Lp(a) levels [157]. Studies investigating the effect of exercise on Lp(a) levels have yielded inconsistent results. While some studies have shown no significant changes, others have reported mild to moderate decreases in Lp(a) levels following exercise. The extent of change in Lp(a) levels may depend on factors such as age, the type, intensity, and duration of physical activity [59,158]. Overall, the impact of lifestyle modifications on Lp(a) levels is not fully understood and may vary among individuals. Future studies are needed to elucidate the mechanisms underlying these relationships and to guide appropriate dietary and lifestyle recommendations.

### 9.2. Traditional Lipid-Lowering Therapies

#### 9.2.1. Statins

Statins regulate the de novo synthesis of cholesterol by competitively inhibiting hydroxymethylglutaryl (HMG) CoA reductase, the rate-limiting enzyme in cholesterol biosynthesis. This inhibition results in a reduction in intrahepatic cholesterol concentration. Additionally, statins upregulate the expression of LDL-R on hepatocytes. As a consequence, there is an increased clearance of LDL particles from the circulation, contributing to the overall reduction of LDL-C levels in the bloodstream [159]. Despite the well-established significant role of statins in reducing both LDL-C and the risk of developing ASCVD, data regarding their efficacy on serum Lp(a) levels remain controversial [149]. The initial hypothesis that statins might reduce serum Lp(a) levels stemmed from the possible involvement of the LDL-R in the catabolism of serum Lp(a). This was observed in patients with HeFH, where treatment with either atorvastatin 80 mg or simvastatin 40 mg significantly lowered Lp(a) levels during a two-year follow-up. However, this reduction in Lp(a) concentration was not associated with changes in CIMT after one or two years of observation, raising questions about the role of Lp(a) in the progression of atherosclerosis [160].

Contrary to these original findings, an analysis of the JUPITER trial found that 20 mg of rosuvastatin daily did not significantly change median Lp(a) levels compared to placebo. However, rosuvastatin was associated with a small but statistically significant shift in the overall Lp(a) distribution toward higher percentiles. Moreover, rosuvastatin demonstrated similar cardiovascular benefits regardless of baseline Lp(a) levels, with no significant interaction [126,161]. Similarly, a post-hoc analysis of the ILLUMINATE trial showed a positive and dose-dependent association between atorvastatin dosage and Lp(a) levels in high cardiovascular-risk patients [162]. Subsequent meta-analyses concluded that statins tend to slightly, if at all, increase Lp(a) levels, with no clinically significant impact on either Lp(a) or the atherosclerotic CVD risk attributed to it. These findings remained consistent across different classes and doses of statins [163,164,165]. Notably, polymorphisms in apo(a)’s KIV2 copy repeats have been shown to influence the response to statin therapy in patients with hypercholesterolemia. In this regard, Yahya et al. demonstrated that statins can significantly increase Lp(a) levels in carriers of small apo(a) molecules, defined as ≤22 K4 repeats (low molecular weight phenotype, LMW), while they observed no significant changes among subjects with a high molecular weight phenotype (HMW) [166].

With most studies accounting for the effect of statins on Lp(a) levels, the real impact of statins on the amount of cholesterol in Lp(a) particles remains a practical issue with major clinical significance. Scanu and Hinman conducted a study to investigate the impact of statin therapy on LDL and Lp(a) cholesterol distribution in 80 hypercholesterolemic subjects with elevated plasma LDL-C (above 100 mg/dL) and Lp(a) (above 10 mg/dL) levels. The participants were assessed both at entry and 8 months after the initiation of statin therapy. Importantly, the researchers aimed to estimate the true LDL and Lp(a) cholesterol values by utilizing the values of Lp(a) protein determined using ELISA, along with an understanding of Lp(a)’s chemical composition. This approach was supplemented with data from immunochemical and ultracentrifugal analyses [167].

The findings of the study revealed that the effect of statins on both Lp(a) protein and Lp(a) cholesterol concentrations was of little or no biological relevance. Despite their effectiveness in reducing LDL-C levels in individuals with additional hyperlipoproteinemia(a), statins achieved major reductions in both total LDL-C and estimated (true) LDL-C concentrations. Consequently, the statin-induced reduction in total plasma LDL-C occurred at the expense of the true LDL component, without a significant effect on Lp(a) cholesterol. This phenomenon also led to a change in the LDL-C/Lp(a)-cholesterol ratio among statin-treated patients [167].

#### 9.2.2. Ezetimibe

Ezetimibe functions by reducing cholesterol intestinal absorption by targeting the Niemann–Pick C1-like1 (NPC1L1) protein [168]. When combined with statins, it has been demonstrated to further decrease LDL-C and increase high-density lipoprotein-cholesterol (HDL-C) compared to statin monotherapy [169]. However, evidence regarding the efficacy of ezetimibe in reducing Lp(a) levels is conflicting. In a meta-analysis of seven randomized controlled trials involving 2337 patients with primary hypercholesterolemia, ezetimibe monotherapy was associated with a statistically significant but small and clinically insignificant reduction of 7.1% in serum Lp(a) levels [170]. On the contrary, a more recent meta-analysis of 10 randomized controlled trials encompassing 5188 patients found that both ezetimibe monotherapy compared to placebo and its combination with statins compared to statin monotherapy did not lead to a significant reduction in Lp(a) concentration [171].

#### 9.2.3. Fibrates

Fibrates have been demonstrated to substantially reduce TGs and increase HDL-C levels, making them a reasonable second-line treatment for mixed dyslipidemia. They exert their actions by activating the nuclear receptor peroxisome proliferator-activated receptor-alpha (PPAR-alpha), which modifies the expression of enzymes involved in lipid metabolism [172]. Recent studies have highlighted the long-known observation that Lp(a) and triglyceride levels have an inverse relationship [173,174]. Hence, fibrates, through their triglyceride-lowering effect, could theoretically lead to an increase in Lp(a) levels in a dose-dependent manner. However, all available data, including a recent meta-analysis [175], converge on the fact that fibrates have no significant effect on Lp(a) levels.

#### 9.2.4. Niacin

Niacin, also known as nicotinic acid or vitamin B3, has been recognized for over 50 years for its broad-spectrum hypolipidemic effects. Its mechanism of action involves reducing triglyceride synthesis and the transport of free fatty acids from adipose tissue to the liver [176]. Additionally, niacin induces the degradation of all ApoB-containing lipoproteins and selectively inhibits the uptake of ApoA-I without affecting its de novo production. This leads to reductions in all apoB-containing lipoproteins, from chylomicrons to LDL-C and Lp(a), while increasing HDL-C levels [177,178]. Niacin’s effect on lowering Lp(a) levels may also involve the mitigation of *LPA* gene expression [179].

Several randomized placebo-controlled clinical trials have shown that therapeutic doses of niacin can reduce Lp(a) levels by an average of 23% [180]. However, despite this promising hypolipidemic effect, niacin has not been associated with benefits in terms of reducing major cardiovascular events, as demonstrated in recent clinical trials [181]. Furthermore, the use of niacin has been linked to clinically significant adverse events, including newly diagnosed or worsened diabetes mellitus, gastrointestinal issues, musculoskeletal problems, skin manifestations, and an increased incidence of infections and bleeding [180]. Considering the lack of cardiovascular benefit and the potential for adverse effects, niacin is not recommended as a therapy specifically for lowering Lp(a) levels [5].

### 9.3. Novel Hypolipidemic Agents

#### 9.3.1. Mipomersen

Mipomersen is a second-generation antisense oligonucleotide that specifically targets and hybridizes with ApoB100 mRNA, leading to its degradation and blocking its translation. This mechanism results in reduced hepatic production of all apoB-containing atherogenic lipoproteins, including Lp(a) [182]. In 2013, the Food and Drug Administration (FDA) approved mipomersen as an adjunct to lipid-lowering therapy and dietary modifications to lower LDL-C, ApoB, and non-HDL-C in patients diagnosed with homozygous familial hypercholesterolemia (HoFH) [183]. Regarding its effect on Lp(a), data from a pooled analysis of four phase 3 randomized controlled trials, involving 382 patients with various types of hyperlipidemia and cardiovascular risk, demonstrated a decrease in Lp(a) levels by a median of 26.4% compared to placebo [184]. Several contemporary and subsequent studies and meta-analyses have confirmed and augmented these findings, showing Lp(a) reductions ranging from 21% to 27.7% [185,186,187].

Nandakumar et al. attempted to elucidate the mechanisms underlying these Lp(a) reductions by enrolling 14 healthy volunteers to receive weekly placebo injections for three weeks followed by weekly injections of mipomersen for seven weeks [188]. In this study, mipomersen achieved a 21% reduction in plasma Lp(a), which was interestingly attributed to a 27% increase in its fractional catabolic rate, without a significant concomitant change in its production rate, although the latter was found to be predictive of Lp(a) reduction in some individuals [189]. Despite the encouraging results mentioned above, mipomersen has been associated with severe adverse events, including hepatotoxicity, angioedema, and injection site reactions, leading to discontinuation in a majority of patients [186,189]. The European Medicines Agency (EMA) rejected the drug in 2012 and 2013 due to concerns regarding its side effects, while the FDA withdrew its approval in 2019 [190,191].

#### 9.3.2. PCSK9 Inhibition

Proprotein convertase subtilisin/kexin type 9 (PCSK9) is a serine protease known for its role in regulating LDL-R levels in hepatocytes [192]. Gain-of-function mutations in the PCSK9 gene have been linked to FH [193], while loss-of-function mutations are associated with lower LDL-C and Lp(a) levels, as well as reduced cardiovascular events and aortic stenosis [194,195]. PCSK9 inhibitors, such as monoclonal antibodies like evolocumab and alirocumab, as well as synthesis inhibitors like inclisiran, work by increasing LDL-R density on hepatocytes, leading to the enhanced clearance of LDL particles and reduced plasma LDL-C levels [196]. These inhibitors have also been shown to decrease Lp(a) levels by 19–27%, although the exact mechanisms behind this effect are not fully understood [127,128,197]. Possible mechanisms include increased LDL-R-mediated clearance, decreased apo(a) production, or reduced assembly of Lp(a) particles due to decreased availability of apolipoprotein B [173].

##### Monoclonal Antibodies against PCSK9

Monoclonal antibodies targeting PCSK9, such as evolocumab and alirocumab, have demonstrated efficacy in lowering Lp(a) levels along with a reduction in the risk of MACE. In the FOURIER and ODYSSEY OUTCOMES trials, which included over 100,000 patient-years of placebo-controlled observation, treatment with PCSK9 monoclonal antibodies resulted in a median reduction of Lp(a) levels by 27% and 23%, respectively [198,199]. Post-hoc analyses of these trials revealed that patients with higher baseline Lp(a) levels experienced greater absolute reductions in Lp(a) and tended to derive greater benefits in major coronary events from PCSK9 inhibition [127]. Additionally, the absolute risk reduction for total cardiovascular events was greater for higher baseline Lp(a) levels, with each −5 mg/dL reduction in Lp(a) predicting a 2.5% relative reduction in cardiovascular events [200,201]. Furthermore, Lp(a) reduction as a result of PCSK9 inhibition was associated with a potential decrease in the risk of PAD and VTE [202].

Interestingly, the clinical efficacy of PCSK9 monoclonal antibodies varied depending on baseline Lp(a) levels, despite similar reductions in LDL-C levels across all quantiles. The relative and absolute clinical benefit of PCSK9 inhibition was more pronounced in patients with elevated baseline Lp(a) levels, suggesting that elevated Lp(a) may be a modifiable risk factor among patients with nominally controlled LDL-C concentrations on statin treatment [173]. Overall, evolocumab and alirocumab have demonstrated significant clinical importance in the management of hypercholesterolemia, particularly in patients with hyperlipoproteinemia(a), as evidenced by numerous clinical trials, randomized controlled trials (RCTs), and meta-analyses beyond the initial studies [128,200,203,204,205].

##### Inclisiran

Inclisiran, a double-stranded small interfering RNA, suppresses PCSK9 mRNA translation, leading to reduced PCSK9 protein synthesis. It is approved by both the EMA and the FDA for lowering LDL-C levels, similar to monoclonal PCSK9 inhibitors [206]. FDA approval in July 2023 extended the use of inclisiran to patients at increased ASCVD risk with elevated LDL-C levels and comorbidities, such as hypertension and T2DM, even without a prior cardiovascular event [8]. Besides its efficacy against LDL-C, several large clinical trials have shown promising results in reducing Lp(a) levels [66].

Preliminary data from a phase 1 trial indicated that inclisiran significantly reduced Lp(a) levels by 48.1% for at least six months [207]. Subsequently, multiple phase 2 and 3 trials, conducted under the ORION trials umbrella, further evaluated inclisiran’s impact on Lp(a) reduction [206]. In the ORION-1 trial, inclisiran effectively lowered Lp(a) levels, although statistical significance was not achieved [208,209]. The ORION-3 trial, an open-label extension of ORION-1, demonstrated Lp(a) reductions of 6.3% and 14.3% in the inclisiran-only arm and switching arm, respectively [210]. Similarly, in the ORION-9 trial involving HeFH patients, inclisiran effectively reduced Lp(a) levels [211]. In ORION-10 and ORION-11 trials, inclisiran achieved significant reductions in Lp(a) levels, although statistical significance was not provided due to the multiplicity of testing [197]. A post-hoc analysis of ORION-11 revealed a placebo-corrected percentage decrease in the Lp(a) levels of 28.5% [212].

Larger outcome trials will provide more conclusive evidence regarding inclisiran’s impact on Lp(a) levels and its ability to reduce MACE. ORION-4, enrolling participants with pre-existing ASCVD, and VICTORION-2 PREVENT, enrolling participants with established CVD, are ongoing phase 3 trials expected to evaluate inclisiran’s effects on MACE reduction [8,66].

#### 9.3.3. Bempedoic Acid

Bempedoic acid acts as an inhibitor of adenosine triphosphate (ATP) citrate lyase, an enzyme upstream of HMG-CoA reductase in the cholesterol biosynthesis pathway. Current literature suggests that bempedoic acid, either as monotherapy or in combination with a statin or ezetimibe, primarily reduces LDL-C levels and, to a lesser extent, other atherogenic lipoproteins [213]. Recent studies have indicated that bempedoic acid can reduce the risk of MACE among statin-intolerant patients requiring modest lipid lowering, despite the presence of side effects such as gout and cholelithiasis [214]. However, regarding Lp(a), there is no reported benefit of bempedoic acid thus far [173]. In a phase 2 trial, bempedoic acid was associated with a slight increase in serum Lp(a), although this was not statistically significant (Esperion Therapeutics, data on file). Similarly, in a recent post-hoc analysis of the CLEAR Harmony trial, the placebo-corrected median percent changes from baseline to 12 weeks associated with bempedoic acid for Lp(a) were 2.4% (0.0 to 4.8) [215]. Furthermore, a randomized, controlled trial of bempedoic acid combined with a PCSK9 inhibitor versus a PCSK9 inhibitor alone demonstrated that, although bempedoic acid significantly lowered LDL-C levels when added to a PCSK9 inhibitor, there was no difference in the change of Lp(a) levels from the baseline between the study arms [216].

#### 9.3.4. CETP Inhibitors

Cholesteryl ester transfer protein (CETP) facilitates the transfer of cholesteryl esters from HDL molecules to ApoB-containing lipoproteins, such as LDL-C, VLDL, and Lp(a) [217]. CETP inhibitors, including torcetrapib, anacetrapib, dalcetrapib, evacetrapib, and obicetrapib (TA-8995), significantly elevate HDL-C levels and decrease Lp(a) levels. Potent CETP inhibitors, excluding dalcetrapib, also lower concentrations of ApoB and LDL-C [218,219]. In the ILLUMINATE trial involving over 15,000 patients receiving atorvastatin treatment, torcetrapib significantly increased HDL subclasses LipoproteinA-I (LpA-I) and LpA-I:A-II equally, along with the ApolipoproteinC-III (ApoC-III) content of HDL, while decreasing Lp(a) levels by approximately 10%. However, the trial was halted prematurely due to torcetrapib being linked to a significant increase in the risk of cardiovascular events and death from any cause, primarily from cancer and infections [162,220].

Anacetrapib has been shown to effectively result in lowering Lp(a) levels, ranging from 34.1% to 43.1% [221,222,223]. Dalcetrapib modestly but significantly reduced Lp(a) levels compared to placebo in an ad hoc analysis of the dal-OUTCOMES trial, while evacetrapib decreased Lp(a) by up to 40% in dose-ranging studies and by 31% in combination with statins [224,225,226]. Obicetrapib, as an adjunct to high-intensity statin therapy, achieved significant reductions in the Lp(a) levels of 33.8% and 56.5% with doses of 5 mg and 10 mg, respectively [227]. Despite their promising lipid-modifying effects, large cardiovascular outcome trials have yielded mixed results. Anacetrapib demonstrated a modestly favorable clinical effect, reducing major vascular events by 9% over 4 years in patients enrolled in the REVEAL trial. However, a meta-analysis of 11 RCTs indicated that CETP inhibitors, including dalcetrapib, anacetrapib, evacetrapib, and obicetrapib, were not associated with an increase in MACE, with a decreasing, non-statistically significant trend observed for non-fatal MI and cardiovascular mortality [228,229]. Recent findings suggest that evacetrapib and torcetrapib may increase HDL-C subspecies associated with a higher risk of CHD, potentially negating the anticipated benefits of CETP inhibition [230]. Due to these mixed cardiovascular outcomes and various safety concerns, CETP inhibitors have not been approved for therapeutic use and are excluded from routine clinical practice [8].

### 9.4. Lipoprotein Apheresis

Lipoprotein apheresis (LA) is a therapeutic technique designed to selectively remove apoB-containing lipoproteins from the bloodstream, including lipoprotein(a) [231]. Beyond its primary role in reducing lipoprotein levels, LA has been found to exert various pleiotropic effects that may contribute to its therapeutic benefits. These effects include reductions in inflammation, improvements in blood viscosity, and enhancements in endothelial function. Additionally, recent evidence suggests that LA may also remove extracellular vesicles (EVs) in individuals with elevated serum Lp(a) levels [232]. Several methods are available for performing LA, including adsorption, precipitation, and filtration techniques. LA is typically indicated for individuals with familial hypercholesterolemia (both heterozygous and homozygous) who fail to achieve therapeutic targets despite maximal tolerated lipid-lowering therapy. However, it is worth noting that a rebound phenomenon can occur following LA, with LDL-C and Lp(a) concentrations returning to baseline levels within approximately two weeks after treatment [231].

While LA has been shown to significantly reduce Lp(a) concentrations, its clinical impact on CVD risk mitigation is still a topic of debate. Available data suggest that LA may improve CVD outcomes, with reported reductions in CVD events ranging from 54% to 90%. In Germany, LA is recommended for individuals with Lp(a) concentrations exceeding 60 mg/dL and progressive CVD, irrespective of LDL-C levels. Data from the German Lipoprotein Apheresis Registry (GLAR) indicate a 72% reduction in Lp(a) levels and a 97% reduction in major CVD events following LA, although these findings are from observational studies and lack randomized controls [233]. The Prospective Pro(a)LiFe study further supports the beneficial effects of LA on CVD outcomes. In this study involving participants with Lp(a) hyperlipoproteinemia and progressive CVD, a single LA treatment led to a 68% reduction in Lp(a) concentrations and a significant decline in the annual rate of CVD events over a five-year period [234]. Similarly, the G.I.L.A. (Gruppo Interdisciplinare Aferesi Lipoproteica) pilot study confirmed the long-lasting cardiovascular benefits of LA [235]. Overall, while LA effectively reduces Lp(a) levels and has shown promise in improving CVD outcomes, further research, including randomized controlled trials, is needed to fully elucidate its clinical efficacy and long-term effects on cardiovascular burden. The effects of lipid-lowering treatments on lipoprotein(a) are detailed in Table 1.

## 10. Molecular Lp(a)-targeting Therapies: In the Heart of the Problem

### 10.1. Pelacarsen

Pelacarsen, formerly known as ISIS 681,257 or IONIS-Apo(a)-LRx, is a second-generation liver-targeted antisense oligonucleotide designed to reduce Lp(a) levels. It is conjugated with a trifurcated N-acetyl-galactosamine (GalNAc) molecule, forming a complex that undergoes endocytosis by hepatocytes through the asialoglycoprotein receptor (ASGPR). Pelacarsen inhibits apolipoprotein(a) formation by preventing mRNA *LPA* translation. The complex is taken up by hepatocytes, leading to ASGPR separation from GalNAc. ASGPR is either recycled or degraded, while GalNAc undergoes degradation in lysosomes. Pelacarsen has demonstrated efficacy in reducing serum Lp(a) levels and has been shown to be a safe approach with minimal adverse events, such as mild injection site reactions [236,237]. Notably, pelacarsen has demonstrated the capability to reduce Lp(a) levels independently of different LPA alleles and isoforms [238].

Recent studies, including a randomized placebo-controlled trial in 27 healthy Japanese individuals, have affirmed the safety and tolerability of subcutaneous pelacarsen. In this study, monthly administration of pelacarsen at 80 mg demonstrated a peak serum reduction in lipoprotein(a) of 106.2% at day 85 in the multiple-dose cohort [239]. The same dosing regimen is currently under investigation in the Lp(a) HORIZON trial, considered a pivotal study for pelacarsen. Lp(a) HORIZON is an ongoing phase 3 multicenter, placebo-controlled trial involving 8325 individuals receiving monthly subcutaneous injections of 80 mg pelacarsen or placebo for 4 to 5 years. The primary objective of the trial is to establish the superiority of pelacarsen over placebo in reducing the risk of expanded MACE in individuals with Lp(a) levels ≥ 90 mg/dL, as well as in subjects with Lp(a) levels ≥ 70 mg/dL and established CVD. The trial is estimated to conclude in mid-2025 [ClinicalTrials.gov identifier (National Clinical Trial number): NCT04023552 A Randomized Double-blind, Placebo-controlled, Multicenter Trial Assessing the Impact of Lipoprotein(a) Lowering with Pelacarsen (TQJ230) on Major Cardiovascular Events in Patients with Established Cardiovascular Disease. [(accessed on 5 June 2023)]; Available online: https://beta.clinicaltrials.gov/study/NCT040235520].

A phase 3 clinical trial is ongoing in Germany, with an anticipated completion date in July 2024. This study involves individuals with established ASCVD, elevated levels of apolipoprotein(a), and Lp(a) exceeding 60 mg/dL undergoing weekly lipoprotein apheresis. Participants are treated with 80 mg subcutaneous pelacarsen per month, and the evaluation focuses on the reduction in the lipoprotein apheresis rate [ClinicalTrials.gov identifier (NCT number): NCT05305664 A Randomized, Double-blind, Placebo-controlled, Multicenter Trial Assessing the Reduction of the Rate of Lipoprotein Apheresis after Treatment with Pelacarsen (TQJ230) Compared to Placebo in Patients with Hyperlipoproteinemia(a) and Established Cardiovascular Disease Undergoing Weekly Lipoprotein Apheresis in Germany. [(accessed on 5 June 2023)]; Available online: https://beta.clinicaltrials.gov/study/NCT053056643].

### 10.2. Olpasiran

Olpasiran, formerly known as AMG 890, represents a groundbreaking siRNA designed to decrease apolipoprotein(a) mRNA specifically in the liver. Upon subcutaneous injection, olpasiran is directed to the liver, where it conjugates to GalNAc. Inside hepatocytes, it assembles into the RNA-induced silencing complex (RISC) and binds to apolipoprotein(a) mRNA, facilitating its degradation. This process leads to the suppression of *LPA* gene expression, resulting in the inhibition of Lp(a) formation. Beyond the reduction of serum Lp(a), olpasiran prescription may also lead to a modest decrease in LDL-C and ApoB, with no significant impact on TGs or HDL-C levels [237]. Moreover, olpasiran may affect oxidized phospholipids on apolipoprotein B100 (OxPL-ApoB), as it has demonstrated a dose-dependent decrease in OxPL-ApoB levels. In particular, patients taking 225 mg of the drug every 12 weeks experienced a mean percent change of 104.7% [240].

Several clinical trials have been conducted or are ongoing to evaluate the safety and efficacy of olpasiran. Phase I (NCT04987320) and phase II (NCT04270760) clinical trials indicated that olpasiran was safe and well-tolerated, with a dose-dependent reduction in Lp(a) levels. In a phase 1 placebo-controlled clinical trial (NCT03626662) involving healthy individuals with varying Lp(a) levels, a single dose of olpasiran resulted in a dose-dependent reduction in Lp(a) concentrations, sustained up to 6 months following drug discontinuation. No major side effects were observed, except for one subject experiencing an injection site reaction [241]. Similar favorable effects on Lp(a) levels were observed in another phase 1 trial, where 27 individuals received a single dose of olpasiran (3, 9, 75, or 225 mg). Olpasiran demonstrated good tolerability and lowered Lp(a) levels in a dose-dependent manner [242]. These positive outcomes led to the design of the OCEAN(a)-DOSE trial, a phase 2 clinical trial involving 281 adults with a history of ASCVD and elevated lipoprotein(a) levels. The trial demonstrated a significant and dose-dependent reduction in serum Lp(a) levels with olpasiran administration, particularly with the 225 mg dose administered every 12 weeks. The most common adverse event observed was painful injection site reactions (ISRs) [243].

The ongoing OCEAN(a)-Outcomes trial (NCT05581303) aims to further investigate the long-term clinical effectiveness and safety of olpasiran. Recently initiated, the phase III Olpasiran Trials of Cardiovascular Events and Lipoprotein(a) Reduction [OCEAN(a)] is expected to conclude in December 2026, enrolling 6000 participants across multiple sites in the United States, Australia, Canada, and Japan. Participants are receiving subcutaneous olpasiran or a placebo every 12 weeks, with eligibility criteria including a mean baseline Lp(a) level of ≥200 nmol/L and a history of ASCVD [237].

### 10.3. SLN360

Similar to olpasiran, SLN360 is a promising N-acetyl galactosamine-conjugated siRNA designed to address Lp(a)-related CVD. The mechanism of SLN360 involves targeting and reducing *LPA* mRNA, ultimately leading to *LPA* knockdown and a subsequent reduction in circulating Lp(a) levels [8]. Preclinical in vitro evidence in cynomolgus and human hepatocytes has demonstrated a significant and sustained reduction in Lp(a) levels of up to 95% for at least 9 weeks following injection, with the peak effect observed at day 21 across all dosing groups. Various subcutaneous doses ranging from 0.1 to 9.0 mg/kg were administered, with the minimally effective dose identified as 0.3 mg/kg. Importantly, no evidence of inflammation, cytokine production, complement stimulation, or micronucleus formation associated with SLN360 use, was found [244,245]. Toxicology studies in rats further supported the tolerability of SLN360 in all doses tested [245].

Preliminary results from a phase 1 single ascending dose trial involving 32 participants with elevated serum Lp(a) levels (>150 nmol/L) and no overt CVD demonstrated that each dose regimen of SLN360 (ranging from 30 to 600 mg) was safely associated with persistent dose-related reductions in Lp(a) levels for at least 150 days following injection. Notably, the highest dose led to a reduction in Lp(a) levels of up to 98% [246]. In January 2023, a phase 2 randomized placebo-controlled trial commenced to further investigate the safety, efficacy, and tolerability of SLN360. This trial involves 160 participants at high ASCVD risk with serum Lp(a) levels exceeding 125 nmol/L. The study is expected to be completed by mid-2024 [247,248].

### 10.4. Lepodisiran

Lepodisiran (LY3819469) is an N-acetyl-galactosamine-conjugated small interfering RNA with a unique tetraloop structure, designed to achieve lasting reductions in lipoprotein(a) synthesis by targeting the production of apolipoprotein(a) by the liver. In a phase 1 clinical trial conducted in the United States and Singapore, 48 subjects without CVD and with Lp(a) levels exceeding 30 mg/dL were enrolled. Participants were randomized to receive either a placebo or a single subcutaneous dose of lepodisiran (4 mg, 12 mg, 32 mg, 96 mg, 304 mg), with the 608 mg regimen involving two injections. Exclusions from the study included individuals under 18 years old, women of reproductive age, adults with a history of smoking or alcohol consumption, and patients with impaired glomerular filtration rate (GFR) or chronic liver disease. The primary outcome of the study focused on the safety and tolerability of lepodisiran use, which proved to be safe and well-tolerated. A single serious adverse event occurred, involving a facial injury following a fall from a bicycle 141 days after prescription. Transient pain at the injection site was observed in subjects across most study groups, including the placebo group. Additionally, a small number of participants experienced an increment in serum transaminases and creatine phosphokinase [249].

Regarding secondary endpoints, the pharmacokinetic and pharmacodynamic effects on serum fasting Lp(a) levels through a maximum follow-up period of 48 weeks were elucidated, including serum concentrations of lepodisiran following 168 days of prescription. Serum levels of the drug increased within the first hour, peaked within 10.5 h, and were eliminated by 48 h following injection. Lepodisiran led to a dose-dependent decrease in serum Lp(a) levels, with a maximal median change exceeding the 90% observed for the three highest doses studied (96, 304, and 608 mg). Remarkably, the therapeutic effect of lepodisiran lasted longer in the 608 mg regimen group. In conclusion, lepodisiran appears to be a safe and well-tolerated treatment, producing dose-dependent, extended-duration reductions in serum Lp(a) concentrations. However, the precise mechanism underlying lepodisiran’s long-term effects remains unclear, and its impact on CVD risk has not been evaluated. Lepodisiran is currently undergoing a phase 2 clinical trial [249].

### 10.5. Muvalaplin

Muvalaplin (LY3473329) stands out as a small molecule designed to inhibit the formation of lipoprotein(a) particles, representing the first oral agent specifically developed to reduce serum Lp(a) levels. The key concept underlying muvalaplin’s function is its potential resemblance to naturally occurring variants in apolipoprotein(a) that cannot interact with ApoB100. This resemblance is believed to lead to a decrease in serum Lp(a) concentrations. Muvalaplin achieves this by acting as a disruptor of the noncovalent interaction between ApoB100 and apo(a), binding to the apo(a) KIV domains 7 and 8 [250].

Recent findings from a phase 1 randomized, placebo-controlled human trial conducted by Nicholls et al. shed light on muvalaplin’s effects on serum Lp(a) levels. The study enrolled 114 participants divided into two groups. The first group included 55 healthy individuals who received a single ascending dose of muvalaplin (ranging from 1 mg to 800 mg). The second group comprised healthy adults with elevated Lp(a) concentrations above 30 mg/dL, receiving multiple single daily doses of muvalaplin (30–800 mg) for a two-week period. Safety, tolerability, pharmacokinetic, and pharmacodynamic parameters were the main outcomes assessed [251].

The study concluded that muvalaplin administration for up to 14 days is a safe and well-tolerated treatment approach, with no major side effects of concern. Peak serum concentrations of muvalaplin occurred 2 to 5 h post-administration, with an elimination half-life ranging from 12 to 67 h. The drug led to a rapid reduction in serum Lp(a) levels within 1 day, further decreasing with repeated dosing. The reduction was dose-dependent, reaching up to 65% with a daily oral prescription regimen for 2 weeks. Notably, the maximum reduction for doses exceeding 100 mg was observed on days 14 and 15, and reduced Lp(a) levels persisted for up to 50 days after the last dose, especially for doses ≥ 300 mg. Muvalaplin did not interfere with plasminogen and did not cause significant changes in other lipid parameters [251].

While muvalaplin showed a lesser degree of reduction in Lp(a) levels compared to parenteral Lp(a)-targeting therapies, its oral administration might offer the potential advantages of lower cost and better compliance. It is important to note that existing immunoturbidometric assays based on anti-apo(a) polyclonal antibodies, which are used to quantify Lp(a) levels, are believed to underestimate muvalaplin’s impact. This is because these methods are presumed to measure not only free apo(a) but also apo(a) bound to muvalaplin in the circulation. Currently, two ongoing trials (NCT05778864 and NCT05563246) focus on muvalaplin’s pharmacokinetics in subjects with renal impairment and its effects on adults with elevated Lp(a) levels at high CVD risk, respectively [250].

### 10.6. CRISPR/Cas9 Lp(a) Genome Editing

**Figure 4:**
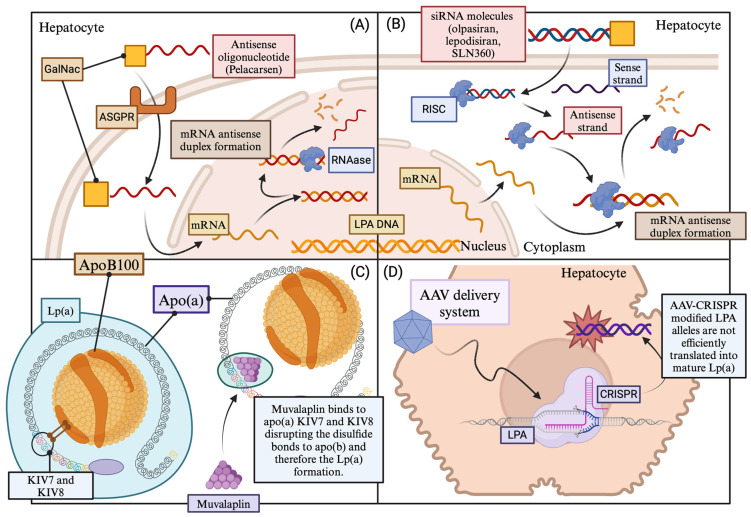
Schematic presentation of the emerging molecular Lp(a)-targeting therapies: (**A**). Pelacarsen is the only ASO that specifically targets the formation of Lp(a). Upon conjugation with GalNAc, the complex undergoes endocytosis into hepatocytes via ASGPR. Subsequently, the translation of mRNA encoding for *LPA* is inhibited, leading to reduced formation of apo(a). (**B**). Once inside the hepatocytes, siRNAs are incorporated into the RISC. Thereafter, they bind to the mRNA encoding for apo(a). This binding triggers the degradation of the apo(a) mRNA, ultimately leading to the inhibition of *LPA* gene expression. (**C**). Muvalaplin binds to the apo(a) KIV7 and KIV8 domains, causing disruption of the noncovalent interaction between apo(a) and ApoB100. As a result, inhibition of the Lp(a) formation is observed (**D**). AAV facilitates the transfer of CRISPR in the liver, where CRISPR disrupts the *LPA* gene, resulting in the removal of Lp(a) particles from the plasma [236,237,238,239,240,241,242,243,244,245,246,247,248,249,250,251,252,253,254]. Abbreviations: AAV: adeno-associated virus; apo(a): apolipoprotein(a); ApoB100: apolipoproteinB100; ASGPR, Asialoglycoprotein receptor; ASO: antisense oligonucleotide; CRISPR: Clustered Regularly Interspaced Short Palindromic Repeat; GalNAc: N-Acetylgalactosamine; KIV: kringle IV; Lp(a): lipoprotein(a); mRNA, messenger RNA; RISC: RNA-induced silencing complex; RNAase: ribonuclease; siRNA: small-interfering RNA. Created with BioRender.com.

In vivo editing of the *LPA* gene using clustered, regularly interspaced palindromic repeats/CRISPR-associated 9 (CRISPR-Cas9) technology holds promise as a potential intervention for reducing CVD risk associated with elevated Lp(a) levels. CRISPR-Cas9 is a powerful gene-editing tool that can precisely target and modify specific genes in living organisms [252]. On the other hand, adeno-associated virus (AAV) vectors have been utilized as delivery vehicles for CRISPR-Cas9 components, as they exhibit high tropism for the liver [253]. Complexing CRISPR-Cas9 with AAV facilitates its delivery to liver cells, enabling targeted gene editing within the liver. In a transgenic mouse model expressing physiological levels of apo(a), CRISPR-Cas9 was employed to permanently disrupt the *LPA* gene in the liver, leading to the removal of Lp(a) from the circulation. In this experimental model, the AAV-CRISPR complex effectively reduced plasma concentrations of apo(a) to almost undetectable levels within a week of administration. This reduction in apo(a) persisted for at least four weeks of observation, with over 99% elimination in male mice and over 96% elimination in female mice [254]. Although of high importance, further research is needed to evaluate the safety, feasibility, and long-term efficacy of this approach in human subjects. Figure 4 provides a schematic presentation of the underlying molecular mechanisms via which the aforementioned agents target lipoprotein(a). Major clinical studies regarding the effects of the Lp(a)-targeting agents on Lp(a) levels are presented in Table 2.

## 11. Conclusions

Hyperlipoproteinemia(a) is a metabolic disorder characterized by elevated levels of lipoprotein(a), which is increasingly recognized as a risk factor for ASCVD. Despite its significance, the exact prevalence and clinical impact of hyperlipoproteinemia(a) remains uncertain, partly due to challenges in standardizing and validating methods for measuring Lp(a) levels. Additionally, the lack of specific and effective pharmacological interventions further complicates the management of this condition. Unlike other types of hyperlipidemia, dietary modifications and lifestyle interventions have limited effects on reducing Lp(a) levels. Traditional lipid-lowering therapies, such as statins, may have modest or negligible effects on Lp(a) levels and do not consistently reduce ASCVD risk associated with hyperlipoproteinemia(a). On the other hand, PCSK9 inhibitors have emerged as promising agents capable of significantly lowering Lp(a) levels while mitigating CVD events. In recent years, various novel molecular therapeutic strategies specifically targeting Lp(a) have been developed, showing impressive reductions in serum Lp(a) concentrations. However, the clinical implications of these reductions in CVD risk remain unclear and require further investigation. Several clinical trials are currently underway, aiming to shed light on the potential effect of these novel pharmaceutical interventions on cardiovascular burden.

## Figures and Tables

**Table 1:** The impact of traditional and novel lipid-modifying agents on Lp(a) levels.

Agent	Author/Study/Year	Impact on Lp(a)	Remarks
Atorvastatin	Arsenault et al., Clinical Trial/2018 [162]	Dose-dependent ↑ of Lp(a) with ↑ atorvastatin doses	T2DM was associated with ↓ Lp(a) levels
Ezetimibe	Sahebkar et al., Systematic review and meta-analysis of randomized controlled trials/ 2018 [171]	No significant possible reduction (0–5%)	The result was confirmed both when ezetimibe was used as monotherapy and in combination with a statin
Fenofibrate	Ko et al., Prospective case-control study/ 2005 [174]	Possible ↑ of Lp(a) levels in patients with hypertriglyceridemia	1. Changes in Lp(a) levels were negatively correlated with changes in triglyceride levels 2. Liver function influenced the effect of fenofibrate on Lp(a)
Niacin	Sahebkar et al., Systematic review and meta-analysis of randomized placebo-controlled trials/2016 [177]	Significant ↓ of Lp(a) levels (WMD: −22.90%, 95% CI: −27.32, −18.48, *p* < 0.001).	No significant association between the changes in plasma concentrations of Lp(a) with niacin dose, treatment duration, and percentage change in plasma HDL-C concentrations
Mipomersen	Reeskamp et al., Randomized Controlled Trial/ 2019 [187]	Significant ↓ of Lp(a) by 27.7%	1. Additional significant ↓ in LDL-C and ApoB 2. Limited tolerability and ↑ hepatic transaminase levels in 21% of patients
Bempedoic acid	Ridker et al., Secondary biomarker analysis of the randomized placebo-controlled multi-center CLEAR Harmony trial /2023 [215]	Median percent change of Lp(a) levels from baseline to 12 weeks was 2.4%	The pattern of lipid lowering and inflammation inhibition with bempedoic acid was almost identical to what is observed with statin therapy
Obicetrapib	Nicholls et al., Clinical trial/2022 [227]	Significant ↓ of Lp(a) by 33.8% and 56.5% with doses of 5 mg and 10 mg, respectively	1. Significant ↓ in LDL-C, non-HDL-C, and ApoB 2. Significant ↑ in HDL-C 3. The most prevalent adverse events were gastrointestinal disorders (primarily nausea) and nervous system disorders (primarily headache).
Evolocumab	O’Donoghue, Randomized controlled trial/ 2019 [127]	Median ↓ of Lp(a) at 48 weeks by 26.9% (6.2−46.7%)	Patients with higher baseline Lp(a) levels experienced greater absolute ↓ in Lp(a) and tended to derive greater coronary benefit
Alirocumab	Szarek et al., Randomized controlled trial/ 2020 [201]	Median Lp(a) ↓ in Lp(a) −5.0 [−13.6, 0] mg/dL	1. ↓ of Lp(a) independently predicted lower risk of total cardiovascular events 2. Each 5 mg/dL ↓ in Lp(a) predicted a 2.5% relative ↓ in cardiovascular events
Inclisiran	Ray et al., Controlled clinical trial/ 2023 [210]	↓ Lp(a) of 6.3% and 14.3% in the inclisiran-only arm and switching arm, respectively	Twice-yearly inclisiran provided sustained ↓ in LDL-C and PCSK9 concentrations and was well tolerated over 4 years in the extension study

Abbreviations: ↑: increased; ↓: reduced; ApoB: apolipoprotein B; HDL-C: high-density lipoprotein-cholesterol; LDL-C: low-density lipoprotein-cholesterol; Lp(a): lipoprotein(a); PCSK9: proprotein convertase subtilisin/kexin type 9; T2DM: diabetes mellitus type 2; WMD: weighted mean difference.

**Table 2:** Major clinical studies showing the effects of novel molecular agents on serum Lp(a) levels.

Authors, Year, Agent	Study’s Characteristics	Results
Karwatowska-Prokopczuk et al., 2023 Pelacarsen [239]	Randomized double-blind, placebo-controlled study: 1. 29 healthy Japanese individuals 2. Effect on serum Lp(a): - SAD: 20 mg, 40 mg, 80 mg - MD: 80 mg monthly (4 doses)	1. Safety and tolerability 2. No serious side effects 3. In the MD cohort: mean serum peak levels of pelacarsen at 4 h 4. In the SAD cohort: max. ↓ of Lp(a) by 73.7% at 80 mg 5. In the MD cohort: max. ↓ of Lp(a) by 106.2% at day 85
Sohn et al., 2022, Olpasiran [242]	Phase I, open-label, parallel-design clinical study enrolling 27 subjects (Japanese/non-Japanese) - Japanese: received olpasiran at 3 mg, 9 mg, 75 mg, 225 mg - Non-Japanese: received olpasiran at 75 mg	1. Safety and tolerability 2. No serious side effects 3. ↓ of Lp(a) as early as day 4 4. ↓ of Lp(a) in a dose-dependent way 5. Mean percentage ↓ of Lp(a) from the baseline: 56–99% 6. Max. ↓ of Lp(a) at day 57 7. The magnitude and durability of ↓ of Lp(a) were similar between 2 groups
Nissen et al. 2022, SLN360 [246]	Phase 1 clinical trial: 1. 32 adults with Lp(a) ≥ 150 nmol/L with no history of CVD 2. Effect on serum Lp(a): SLN360 vs. placebo at 30 mg, 100 mg, 300 mg, 600 mg	1. Safety and tolerability 2. No significant side effects 3. Dose-dependent ↓ Lp(a) 4. Median Lp(a) concentrations were ≥70% and ≥80% below baseline at day 150 following administration of the 300 mg and 600 mg SLN360 doses, respectively. 5. ↓ of Lp(a) persisted for at least 150 days after administration
Nissen et al. 2023, Lepodisiran [249]	Phase 1 clinical trial: 1. 48 subjects with Lp(a) ≥ 30 mg/dL without CVD. 2. Effect on serum Lp(a): Lepodisiran vs. placebo at 4 mg, 12 mg, 32 mg, 96 mg, 304 mg, and 608 mg 3. Excluded from the study: - Age < 18 years old - Women of reproductive age - Smoking, alcoholism - Chronic renal failure or liver disease	1. Safety and tolerability 2. Side effects: - Most common: transient pain at the injection site - ↑ SGOT, SGPT, CPK (mild) 3. Peak lepodisiran’s levels at 10.5 h 4. Undetectable levels of lepodisiran by 2 days 5. Dose-dependent ↓ of Lp(a) with ↓ 97% reduction of Lp(a) in the 608- mg dose group 6. The treatment effect lasted longer in the 608 mg dose group
Nicholls et al. 2023, Muvalaplin [251]	Phase 1 randomized placebo-controlled human study: 1. 114 healthy individuals aged 18–69 years old with BMI ≤ 30 kg/m^2^: - 1st part of the study: The effect of 1 dose (1–800 mg) of muvalaplin vs. placebo on serum Lp(a) - 2nd part of the study: The effect of 1 dose (30–800 mg) of muvalaplin vs. placebo taken over 14 days in subjects with Lp(a) ≥ 30 mg/dL	1. Safe and well-tolerated for up to 14 days of prescription 2. No significant side effects 3. Peak serum levels of muvalaplin 2–5 days post-administration 4. Rapid ↓ of Lp(a) within 1 day 5. Max. ↓ of Lp(a) 63–65% for doses ≥ 100 mg on days 14 and 15 6. Sustained ↓ in Lp(a) levels for up to 50 days, particularly for doses ≥ 300 mg 7. No significant changes in PLG

Abbreviations: ↑: increased; ↓: reduced; BMI: body mass index; CPK: creatinine phosphokinase; Lp(a): lipoprotein(a); MDs: multiple doses; PLG: plasminogen; SADs: single ascending doses; SGOT: Serum Glutamic-Oxaloacetic Transaminase; SGPT: Serum Glutamic Pyruvic Transaminase.

## Data Availability

Not applicable.

## Abbreviations

AAV vectors, adeno-associated virus vectors; ARIC: Atherosclerosis Risk in Communities; ATP, adenosine triphosphate; ASE, American Society of Echocardiography; ASO, antisense oligonucleotide; AVS, aortic valve stenosis; Apo(a), Apolipoprotein(a); ApoB100, Apolipoprotein B100; ApoC-III, Apolipoprotein C-III; ASGPR, asialoglycoprotein receptor; ASCVD, atherosclerotic cardiovascular disease; CAD, coronary artery disease; CAVD, calcific aortic valvular disease; CVD, cardiovascular disease; CETP, cholesteryl ester transfer protein; CIMT, carotid intima-media thickness; CRISPR/Cas9, clustered regularly interspaced palindromic repeats/CRISPR associated 9; CHD, coronary heart disease; COVID-19, Coronavirus disease 2019; DVT, deep vein thrombosis; ELISA, enzyme-linked immunosorbent assay; EMA, European Medicines Agency; EVs, extracellular vesicles; FDB, familial defective ApoB-100; FH, Familial hypercholesterolemia; FDA, Food and Drug Administration; GalNAc, N-Acetylgalactosamine; GLAR, German Lipoprotein Apheresis Registry; GFR, glomerular filtration rate; HeFH, heterozygous familial hypercholesterolemia; HDL, high-density lipoprotein; HDL-C, high-density lipoprotein cholesterol; hs-CRP, high-sensitive C-reactive protein; HoFH, homozygous familial hypercholesterolemia; HMG-CoA, hydroxy-methylglutaryl-coenzyme A; HMW, high molecular weight; ICAM-1, Intercellular Adhesion Molecule 1; IDL, intermediate-density lipoproteins; ISR, injection site reaction; IL-1β, Interleukin 1β; IL-6, Interleukin-6; IL-8, Interleukin 8; KIV, kringle IV; LCAT, lecithin-cholesterol acyltransferase; Lp(a), Lipoprotein(a); LpA-I, Lipoprotein A-I; LA, Lipoprotein apheresis; LMW, low molecular weight; Lp-PLA2, Lipoprotein-associated phospholipase A2; LC-MS/MS, liquid chromatography–tandem mass spectrometry; LDL, low-density lipoprotein; LDL-C, low-density lipoprotein-cholesterol; LDL-R, low-density lipoprotein-receptor; Mac-1, Macrophage 1; MACE, major adverse cardiovascular events; MI, myocardial infarction; MMPs, matrix metalloproteinases; mRNA, messenger RNA; MCP, monocyte chemoattractant protein; MUFA, monounsaturated fatty acids; MI, myocardial infarction; GalNAc, N-acetyl-galactosamine; NCT, National Clinical Trial; NPC1L1, Niemann–Pick C1-like1; OxLDLs, oxidized LDLs; OxPLs, oxidized phospholipids; OxPL-ApoB, oxidized phospholipids on apolipoprotein B; OxPL-LDL, oxidized phospholipids on LDL; PAD, peripheral artery disease; PPAR-alpha, peroxisome proliferator-activated receptor-alpha; PLG, plasminogen; PAI-1, plasminogen activator inhibitor-1; PCSK9, proprotein convertase subtilisin/kexin type 9; PE, pulmonary embolism; RCTs, randomized controlled trials; RNA, ribonucleic acid; RISC, RNA-induced silencing complex; RNAi, RNA interference; siRNA, short interfering RNA; SNPs, single nucleotide polymorphisms; tPA, tissue plasminogen activator; SFA, saturated fatty acids; TG, triglycerides; TLR, toll-like receptor; TNF-α, tumor necrosis factor α; UV, unesterified cholesterol; VCAM-1, vascular cell adhesion molecule 1; VSMCs, vascular smooth muscle cells; VTE, venous thromboembolism; VLDL, very low-density lipoprotein; WHO/IFCCLM, World Health Organization/International Federation of Clinical Chemistry and Laboratory Medicine.
